# 

**Published:** 1853-06

**Authors:** 


No. I.] JUNE. [Vol. IX.

### BUFFALO MEDICAL JOURNAL

AND

### MONTHLY REVIEW

OF

### Medical and Surgical Science.

EDITED BY

AUSTIN FLINT, M. D.

AND

S. B. HUNT, M. D.

BUFFALO:

PUBLISHED BY JEWETT, THOMAS & CO.

Commercial Advertiser Buildings.

1853.

PRICE $2,50 PER ANNUM..........IN ADVANCE.

